# Aldosterone enhances high phosphate–induced vascular calcification through inhibition of AMPK‐mediated autophagy

**DOI:** 10.1111/jcmm.15813

**Published:** 2020-11-04

**Authors:** Jing‐Wei Gao, Wan‐Bing He, Chang‐Ming Xie, Ming Gao, Lei‐Yu Feng, Zhao‐Yu Liu, Jing‐Feng Wang, Hui Huang, Pin‐Ming Liu

**Affiliations:** ^1^ Department of Cardiology Sun Yat‐sen Memorial Hospital of Sun Yat‐sen University Guangzhou China; ^2^ Cardiovascular Department The Eighth Affiliated Hospital Sun Yat‐sen University Shenzhen China; ^3^ Department of Radiology Sun Yat‐sen Memorial Hospital of Sun Yat‐sen University Guangzhou China; ^4^ Department of Cardiology The First Affiliated Hospital of Zhengzhou University Zhengzhou China; ^5^ Medical Research Center Sun Yat‐sen Memorial Hospital of Sun Yat‐sen University Guangzhou China

**Keywords:** aldosterone, autophagy, phenotypic switch, vascular calcification, vascular smooth muscle cell

## Abstract

It remains unclear whether the necessity of calcified mellitus induced by high inorganic phosphate (Pi) is required and the roles of autophagy plays in aldosterone (Aldo)‐enhanced vascular calcification (VC) and vascular smooth muscle cell (VSMC) osteogenic differentiation. In the present study, we found that Aldo enhanced VC both in vivo and in vitro only in the presence of high Pi, alongside with increased expression of VSMC osteogenic proteins (BMP2, Runx2 and OCN) and decreased expression of VSMC contractile proteins (α‐SMA, SM22α and smoothelin). However, these effects were blocked by mineralocorticoid receptor inhibitor, spironolactone. In addition, the stimulatory effects of Aldo on VSMC calcification were further accelerated by the autophagy inhibitor, 3‐MA, and were counteracted by the autophagy inducer, rapamycin. Moreover, inhibiting adenosine monophosphate‐activated protein kinase (AMPK) by Compound C attenuated Aldo/MR‐enhanced VC. These results suggested that Aldo facilitates high Pi‐induced VSMC osteogenic phenotypic switch and calcification through MR‐mediated signalling pathways that involve AMPK‐dependent autophagy, which provided new insights into Aldo excess‐associated VC in various settings.

## 1 INTRODUCTION

Vascular calcification (VC) is a strong predictor of cardiovascular morbidity and mortality, and is linked to ageing, diabetes and chronic kidney diseases (CKD).^1^ Growing evidence now suggests that VC is an actively regulated process resembling bone remodelling, including both inductive and inhibitory processes.^2^ Abnormal activation of the renin‐angiotensin‐aldosterone system plays an important role in the development of cardiovascular diseases, among which aldosterone (Aldo) is a major effector.^3^ Aldo, a mineralocorticoid hormone, binds to mineralocorticoid receptor (MR) and then activates specific intracellular genomic pathways, thus regulating the homeostasis of the cardiovascular system. Once Aldo is overactivated, it can promote vascular oxidative stress, inflammation and apoptosis,^4^ leading to an increased risk of target‐organ damage.^5^ Previous studies suggested that a MR inhibitor, spironolactone (Spiro) ameliorated CKD‐related VC, which indicated the involvement of Aldo in VC.^6^, ^7^, ^8^, ^9^ Besides Aldo excess, a series of mineral disorders and hormonal imbalance including altered homeostasis of calcium, phosphorus, 1,25‐dihydroxyvitamin D and parathyroid hormone, occurs gradually with the decline in kidney function.^10^ Therefore, whether Aldo is an initiator or just a promoter of VC remains unclear^11^ and warrants further study. Primary aldosteronism (PA) is an endocrine disorder characterized by autonomous Aldo secretion and hypertension. Our recent study showed that PA patients, matched for age, sex and blood pressure, exhibited more severe abdominal aortic calcification than those with essential hypertension.^12^ Notably, we noticed that most included PA patients had different degree of reduced estimated glomerular filtration rate, which was tightly linked to increased inorganic phosphate (Pi) concentration.^13^ Therefore, whether the calcified mellitus induced by high Pi is required for Aldo‐enhanced VC, and the potential mechanisms for Aldo‐MR complex to promote VC need to be clarified.

It has been implicated that autophagy acts as a mediator in hyperaldosteronism‐induced target‐organ damage.^14^, ^15^, ^16^ Autophagy is a process highly regulated by adenosine monophosphate‐activated protein kinase (AMPK) signalling; impaired autophagy activity in vascular cells is closely associated with vascular disorders.^17^ Under various pathophysiological conditions, vascular smooth muscle cells (VSMCs) undergo autophagy and generate double‐membrane vesicles called autophagosomes, which fuse with the lysosome leading to lytic degradation of autophagosomal contents.^18^ Recent evidence indicated that up‐regulation of autophagy counteracted the high Pi‐induced phenotypic switch and calcification of VSMCs.^19^, ^20^ However, the role of VSMC autophagy in the VC regulated by Aldo‐MR complex is unknown.

In the present study, we assessed the necessity of high Pi in the association between Aldo and VC in vivo and in vitro. We then explored the roles of AMPK signalling‐dependent autophagy in the Aldo‐enhanced VSMC osteogenic differentiation and calcification.

## 2 MATERIALS AND METHODS

### 2.1 Reagents and antibodies

Foetal bovine serum (FBS) and Dulbecco's Modified Eagle's medium (DMEM) were obtained from Gibco‐BRL (Thermo Fisher Scientific, Inc). High‐phosphate diet (HPD) and standard chow were purchased from Guangdong Medical Laboratory Animal Center. Aldo (A9477), NaH_2_PO_4_ (94046) and Spiro (S1200000) were purchased from Sigma‐Aldrich. The autophagosome inhibitor, 3‐methyladenine (3‐MA) and agonist (rapamycin, Rapa), the autophagic flux inhibitors, bafilomycin A1 (Baf) or chloroquine (CQ), AMPK inhibitor (Compound C) were from Selleck Chemicals.

Antibodies against glyceraldehyde‐3‐phosphate dehydrogenase (GAPDH; #2118), alpha smooth muscle actin (α‐SMA; #19245), runt‐related transcription factor 2 (Runx2; #12556), LC3 (#4599), P62 (#5114), Beclin‐1 (#3495), total AMPKα (T‐ AMPKα; #5831) and phospho‐AMPKα (P‐AMPKα; #50081) were from Cell Signaling Technology Inc. Antibodies against bone morphogenetic protein 2 (BMP2; ab14933), smooth muscle 22 alpha (SM22α; ab10135) and smoothelin (ab204305) were from Abcam. An antibody against osteocalcin (OCN; sc‐30044) antibody was purchased from Santa Cruz Biotechnology.

### 2.2 Animal experiments

Animal experiments were approved by the Committee on Ethics of Animal Experiments, Sun Yat‐sen University (Guangzhou, China) and conducted in accordance with the Guidelines for the Care and Use of Laboratory Animals published by the US National Institutes of Health (NIH Publication No. 85‐23, revised 1996).

Eight‐week‐old female dilute brown non‐agouti 2 (DBA/2N) mice were purchased from Vital River Laboratory Animal Technology Co., Ltd and were randomly divided into four groups: control, HPD, desoxycorticosterone acetate (DOCA) and DOCA with HPD (DOCA + HPD), n = 10 per group. DBA/2N mice are susceptible to VC when exposed to increased oral phosphorus loads independent of impaired kidney function.^21^ HPD containing 2% phosphorus was administered in HPD and DOCA + HPD groups, while the control and DOCA mice were fed standard chow containing 0.7% phosphorus. For DOCA and DOCA + HPD mice, a sustained release tablet (50 mg/21 days, Innovative Research of America) were implanted subcutaneously for 3 weeks from the beginning to mimic the chronic Aldo exposure,^22^ while control and HPD mice were just given sham‐operation.

After 12 weeks, the animals were killed and mouse serum was collected to determine the blood urea nitrogen (BUN), creatinine (Cr), calcium and phosphorus. The thoracic aortas were excised for further analysis.

### 2.3 Cell cultures

Primary mouse VSMCs were extracted from 8‐week‐old male C57BL/6 mice. Briefly, the mice were killed by sodium pentobarbital, and the thoracic aorta was dissected out quickly. After removing the adventitia and intima, the artery segment was cut into 1‐2 mm^2^ sections and placed in a cell culture flask with DMEM containing 4.5 g/L glucose supplemented with 20% FBS, 100 U/mL penicillin and 100 mg/mL streptomycin (growing medium). After confirming the VSMC phenotype and purity by positive immunostaining for α‐SMA (Figure S1), VSMCs were incubated at 37°C in a humidified atmosphere containing 5% CO_2_ and generally switched into DMEM with 5% FBS. VSMCs at passages 4‐8 were used for the experiments. The culture medium was replaced every 3 days. As shown in Figure S2, 2.6 mmol/L NaH_2_PO_4_ was the minimal concentration to induce VSMC calcification (Figure S2).

### 2.4 von Kossa staining and Alizarin red S staining

For von Kossa staining, 3 μm paraffin‐embedded artery sections were exposed to a 5% silver nitrate solution and were placed under an ultraviolet light for 2 hours after the standard dewaxing procedure.

For Alizarin Red staining, VSMCs in 6‐well plates were washed 3 times with PBS after indicated treatments and then fixed with 4% paraformaldehyde for 30 minutes. Then, the cells were washed 3 times with PBS and exposed with 2% Alizarin Red solution (Sigma) for 10 minutes. Positively stained cells display a red‐orange colour.^19^

### 2.5 Alkaline phosphatase (ALP) activity and calcium content detection

The ALP Assay Kit (A059‐2) and Calcium Assay Kit (C004‐2) were from Nanjing Jiancheng Bioengineering Institute. The ALP activity and calcium content were determined according to the manufacturer's instructions. The results were then normalized by the protein content determined by the BCA Protein Assay Kit (P0012) from Beyotime.^23^

### 2.6 Western blotting analysis

The protein samples were extracted from VSMCs with RIPA lysis buffer (Beyotime) on ice for 30 minutes. After centrifugation at 12 400 *g* at 4°C for 10 minutes, the supernatants of VSMCs were harvested to determine the protein expression. The protein samples were mixed with the loading buffer and boiled at 95°C for 5 minutes. The boiled samples were separated on the SDS‐polyacrylamide gels, and the proteins were transferred to the polyvinylidenedifluoride membranes. These membranes were then incubated successively with 5% bovine serum albumin and the primary antibodies: Antibodies against GAPDH (1:1000 dilution), Runx2 (1:500 dilution), BMP2 (1:500 dilution), OCN (1:200 dilution), α‐SMA (1:1000 dilution), SM22α (1:1000 dilution), smoothelin (1:1000 dilution), LC3 (1:1000 dilution), P62 (1:1000 dilution) and Beclin‐1 (1:1000) overnight. The membranes were then washed with TBS‐T, followed by an incubation with a horseradish peroxidase‐conjugated secondary antibody (1:5000 dilution, CST) for 2 hours at room temperature. Protein blot bands were detected by ECL blotting substrate and then exposed to an X‐ray film. The densities of the bands were semi‐quantified and normalized against GAPDH by Image J software (Thermo Scientific).

### 2.7 Autophagy detection using mRFP‐GFP adenoviral vector

mRFP‐GFP‐LC3 double‐labelled adenovirus was purchased from HanBio Technology Co. Ltd., and adenoviral infection was performed according to the manufacturer's instructions. Isolated adult mouse VSMCs were plated in 6 orifice plates and infected after reaching 50%‐70% confluence. Cells were then transduced with adenovirus by DMEM incubating in DMEM supplemented with 2% FBS for 4 hours at 37°C. After infection, VSMCs were then grown in medium with 10% FBS and used for further experiments. Autophagy was observed through a fluorescence microscope (Nikon Eclipse Ti‐s). Autophagic flux was determined by evaluating the number of GFP and RFP puncta (puncta/cell were counted).^24^

### 2.8 Cell viability

Cell viability was measured by Cell Counting Assay Kit‐8 (CCK‐8, Dojindo Molecular Technologies, MD) as described.^25^ In brief, VSMCs were seeded in 96‐well plates at a density of 1‐2 × 10^4^ cells mL^−1^ for 24 hours, following which they were transfected with different multiplicity of mRFP‐GFP adenovirus for 4 hours. 10 µL CCK‐8 solution was then added to each well for 2 hours, and absorbance at 450 nm was measured using a microplate reader (Bio‐Tek). Cell viability was normalized to control.

### 2.9 Statistical analysis

All experiments were independently repeated at least 3 times. Data were presented as mean ± SEM. Differences of data among multiple groups were determined by one‐way ANOVA followed by a LSD test. Statistical analysis was performed with SPSS version 20 (SPSS, Inc, Chicago, IL), and *P* < .05 was considered statistically significant. GraphPad Prism 8.0 was used to draw figures.

## 3 RESULTS

### 3.1 Aldo facilitates calcium deposition in vivo and in vitro

As shown in Table 1, serum phosphorus and BUN levels (both *P* < .05) were higher in HPD‐treated mice than the control and DOCA‐treated mice. However, the serum levels of calcium and Cr were similar among the 4 groups.

**TABLE 1:** Serum biochemical parameters

	Control (n = 10)	DOCA (n = 10)	HPD (n = 10)	HPD + DOCA (n = 10)
BUN (mmol/L)	7.89 ± 0.90	9.71 ± 2.53	12.58 ± 2.69*	14.86 ± 3.54*
Cr (μmol/L)	8.35 ± 1.32	10.93 ± 4.52	11.21 ± 2.77	11.75 ± 3.19
Calcium (mmol/L)	2.14 ± 0.12	2.13 ± 0.08	2.14 ± 0.10	2.19 ± 0.15
Phosphorus (mmol/L)	6.51 ± 0.45	6.48 ± 0.50	11.75 ± 2.34*	12.30 ± 1.21*

Note

Values are means ± SEMs (n = 10).

Abbreviations: BUN, blood urea nitrogen; Cr, creatinine; DOCA, desoxycorticosterone acetate; HPD, high‐phosphate diet.

* *P* < .05 vs control and DOCA groups.

**FIGURE 1:**
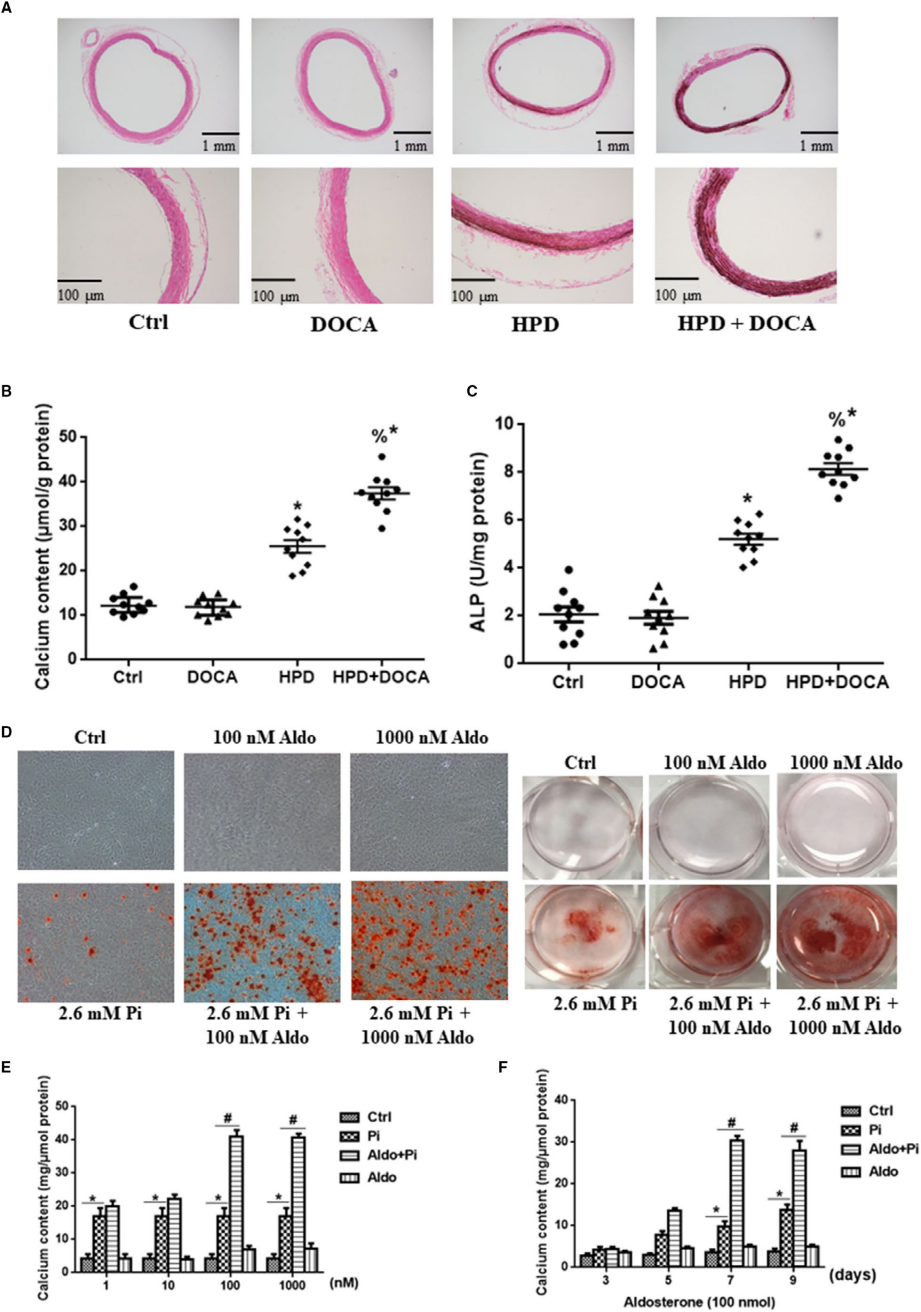
Aldosterone (Aldo) promoted vascular calcification only in the presence of high phosphate (Pi) in vivo and in vitro. A, Von Kossa staining of mouse thoracic aortas (scale bar: 100 μm). B, Calcium content of the thoracic aortas was measured and normalized to the protein levels. C, Quantitative analysis of alkaline phosphatase (ALP) activity in the aortas normalized to the protein content. D, Alizarin red S staining including whole well and microscopic (scale bar, 50 μm) images in rat VSMCs treated with Aldo at different doses plus high Pi or normal Pi stimulation for 7 d. E and F, Calcium content of VSMCs after Aldo induction at different doses (1, 10, 100 and 1000 nmol/L) and timepoints (3, 5, 7 and 9 d) with or without high Pi mellitus (2.6 mmol/L NaH_2_PO_4_). n = 10 per group for in vivo and n = 5 per group for in vitro experiments. *P < .05 vs control group; ^&^ *P* < .05 vs high‐phosphate diet (HPD) group; ^#^ *P* < .05 vs Pi group

To assess vascular medial calcification in HPD‐induced non‐CKD mice, von Kossa staining was performed. As shown in Figure 1A, HPD‐treated mice developed more severe medial calcification than the control mice, and DOCA supplement further aggravated this procalcific effect. However, DOCA alone failed to induce VC. Furthermore, ALP activity (Figure 1B) and calcium content (Figure 1C) analysis of mouse artery tissues also demonstrated that DOCA administration promoted the degree of calcification in vivo only in the presence of HPD treatment (*P* < .05). Consistently in vitro experiments, as proven by alizarin red S staining and the calcium content assay, Aldo could not induce VSMC calcification in medium without high Pi, whereas Aldo accelerated VSMC calcification in the medium containing high Pi in dose‐ and time‐dependent manners (Figure 1D,F; *P* < .05). Thus, we chose the treatment concentration (100 nmol/L) and duration (7 days) of Aldo for the following experiments.

### 3.2 Aldo promotes oseteogenic phenotypic switch of VSMC in MR‐dependent manner in vitro

**FIGURE 2:**
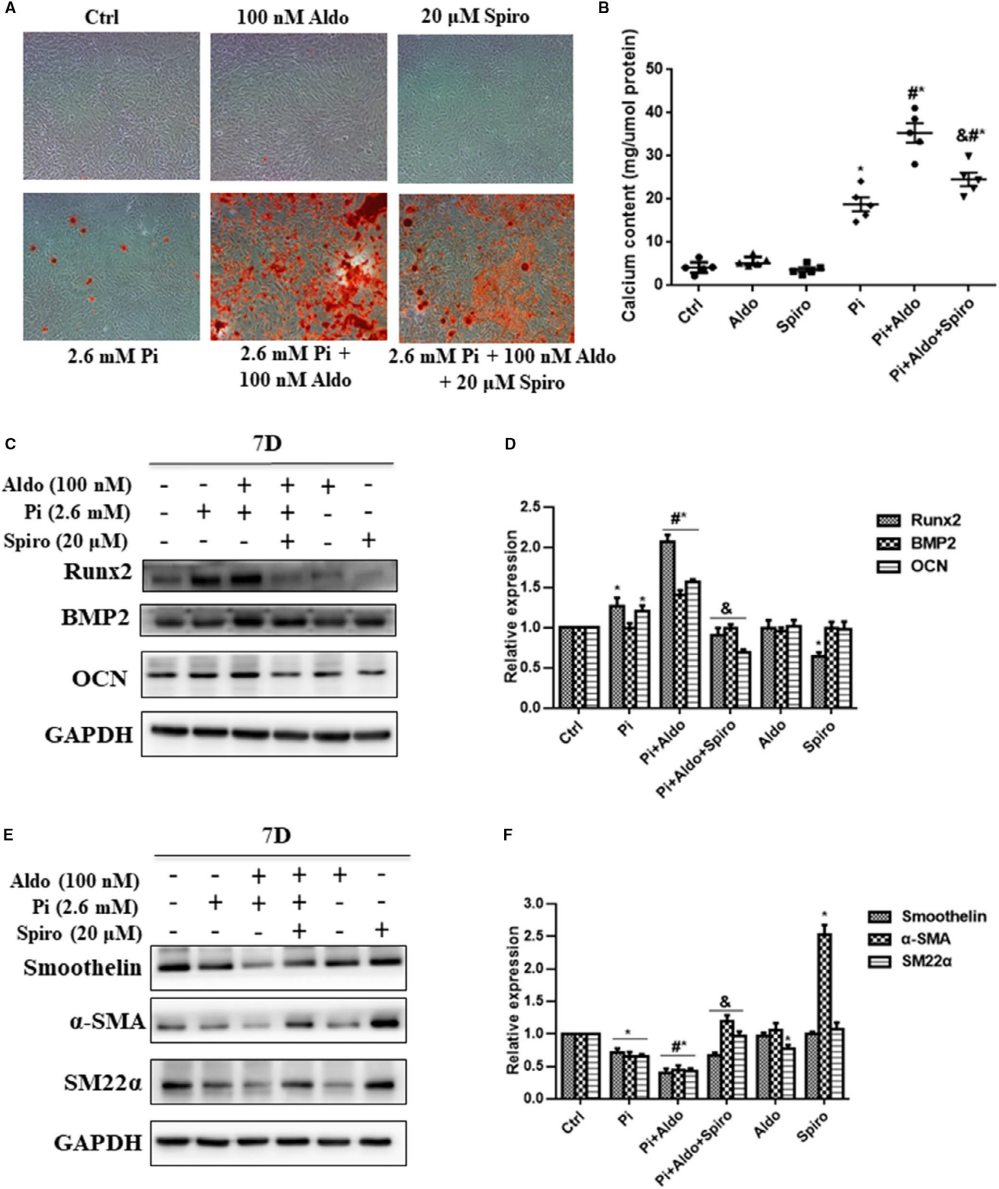
Aldosterone (Aldo) accelerated high phosphate (Pi)–induced vascular smooth muscle cell (VSMC) calcification and phenotypic switch in a mineralocorticoid receptor‐dependent manner. A, Alizarin red S staining of VSMCs. B, Quantification of calcium deposition of VSMCs normalized to the protein content. C and D, Representative Western blot bands and semiquantitative analysis of runt‐related transcription factor 2 (Runx2), bone morphogenetic protein‐2 (BMP2) and osteocalcin (OCN) in VSMCs; E and F, Representative Western blot bands and semiquantitative analysis of smoothelin, alpha smooth muscle actin (α‐SMA) and smooth muscle 22 alpha (SM22α) in VSMCs after induction with different stimuli for 7 d, and 100 nmol/L Aldo and 20 μmol/L spironolactone (Sipro) were used. n = 5 per group. *P < .05 vs control group; ^#^ *P* < .05 vs Pi group; ^&^ *P* < .05 vs Aldo + Pi group

The switch from contractile VSMCs into osteo‐/chondrocytic‐like cells has been increasingly accepted as a key mechanism of VC.^26^ Thus, we attempted to investigate whether Aldo exerted procalcific effects via triggering VSMC phenotypic transdifferentiation. As shown in Figure 2A,B, the procalcific effect of Aldo was greatly suppressed by cotreatment with Spiro (20 μmol/L). The expression levels of osteogenic markers, including Runx2, BMP2 and OCN, were significantly increased in VSMCs treated with high Pi (2.6 mmol/L NaH_2_PO_4_) compared to those in controls (Figure 2C,D; *P* < .05), whereas the expression of contractile proteins, smoothelin, α‐SMA and SM22α, was markedly reduced (Figure 2E,F; *P* < .05). Addition of Aldo further aggravated these changes induced by high Pi. However, cotreatment with Spiro greatly abrogated these effects by Aldo (Figure 2; *P* < .05).

### 3.3 Aldo down‐regulates high Pi‐induced VSMC autophagy

**FIGURE 3:**
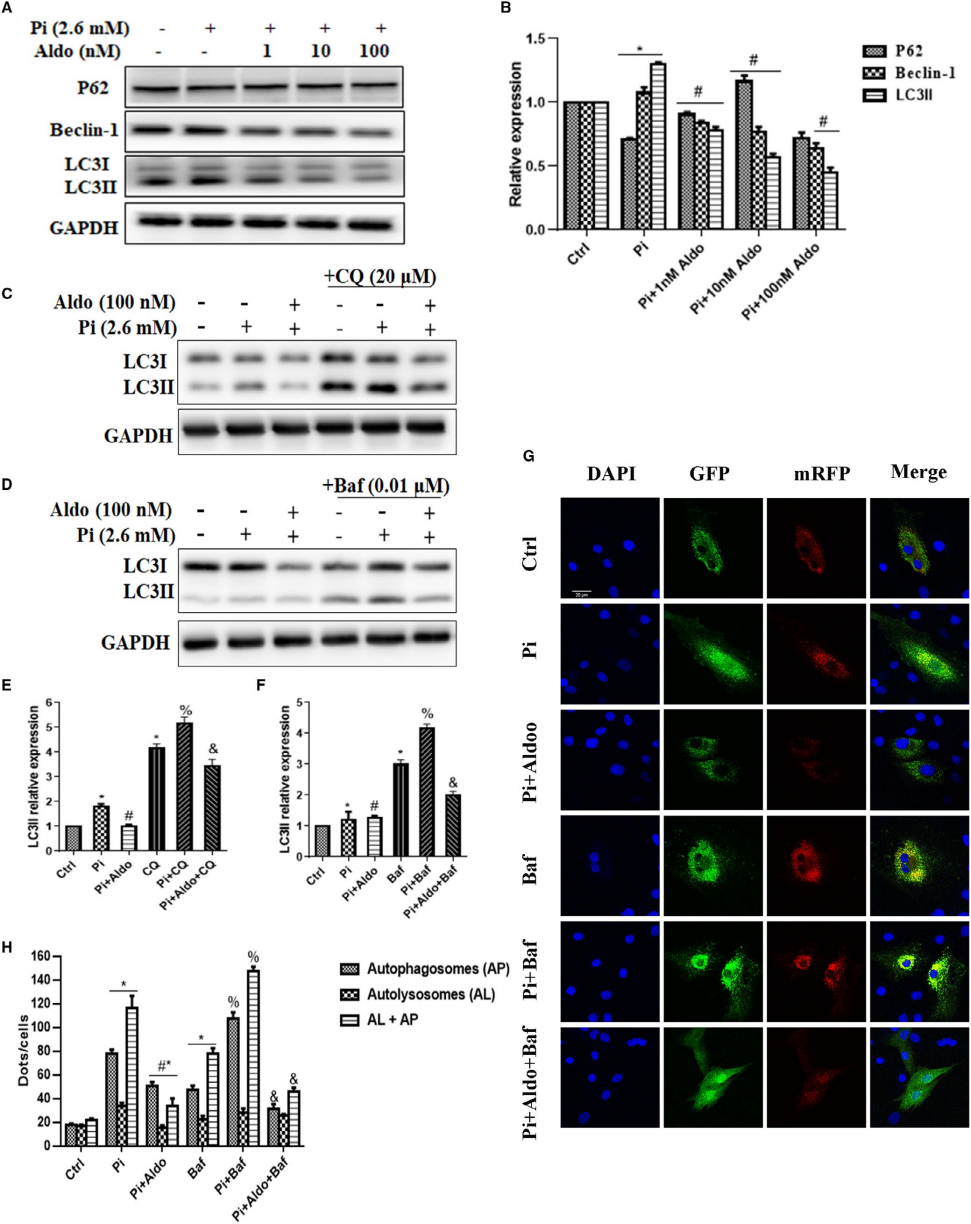
Aldosterone (Aldo) inhibited vascular smooth muscle cell (VSMC) autophagosomes formation. A and B, Representative Western blot bands and semiquantitative analysis of P62, Beclin‐1 and LC3 after Aldo stimulation at different doses for 7 d in the high phosphate (Pi) condition; C‐F, Representative Western blot bands and semiquantitative analysis of LC3 in untreated and Aldo‐treated cells. Note that chloroquine (CQ) (20 µmol/L) or bafilomycin A1 (Baf) (0.01 µmol/L) was added 3 h before Aldo. G and H, Representative confocal images (scale bar, 20 μm) and quantitative analysis of mRFP‐GFP‐LC3 puncta in VSMCs. Bar graph showed the mean number of autophagosomes (yellow dots) and autolysosomes (red dots) per cell. n = 5 per group. *P < .05 vs control group; ^#^ *P* < .05 vs Pi group; ^%^ *P* < .05 vs CQ or Baf group; ^&^ *P* < .05 vs Pi + CQ or Pi + Baf group

We then explored whether Aldo affected VSMC autophagy in high Pi condition. As shown in Figure 3A,B, high Pi treatment induced a significant increase in LC3‐II formation, an indicator of autophagy. However, Aldo stimulation for 7 days caused a dose‐dependent decrease in LC3‐II formation and Beclin‐1 protein expression and reached a peak inhibition at the dose of 100 nmol/L (*P* < .05).

As the change of LC3II is possibly due to modulation of autophagosome formation or autophagic degradation,^27^ we inhibited the lysosomal degradation of LC3‐II by treating cells with Baf (0.01 μmol/L) or CQ (20 μmol/L). Incubated VSMCs with Baf or CQ increased LC3II abundance, which was further increased in high Pi treatment. However, this effect was significantly inhibited by addition of Aldo (Figure 3C‐F; *P* < .05). To obtain further evidence for the specific steps of autophagy inhibited by Aldo, VSMCs were infected with mRFP‐GFP‐LC3 adenovirus (MOI = 100), which did not significantly affect VSMC viability (Figure S3). As shown in Figure 3G,H, there was a considerable increase in GFP/RFP double‐positive autophagosomes (yellow) and RFP‐positive autolysosomes (red) in Baf‐treated cells and this was further increased in the presence of high Pi (*P* < .05). However, addition of Aldo significantly reduced the number of yellow dots but not the red ones. Taken together, these results indicated that Aldo inhibited the formation of autophagosomes.

### 3.4 Aldo‐progressed VSMC calcification and osteogenic differentiation are through autophagy inhibition

**FIGURE 4:**
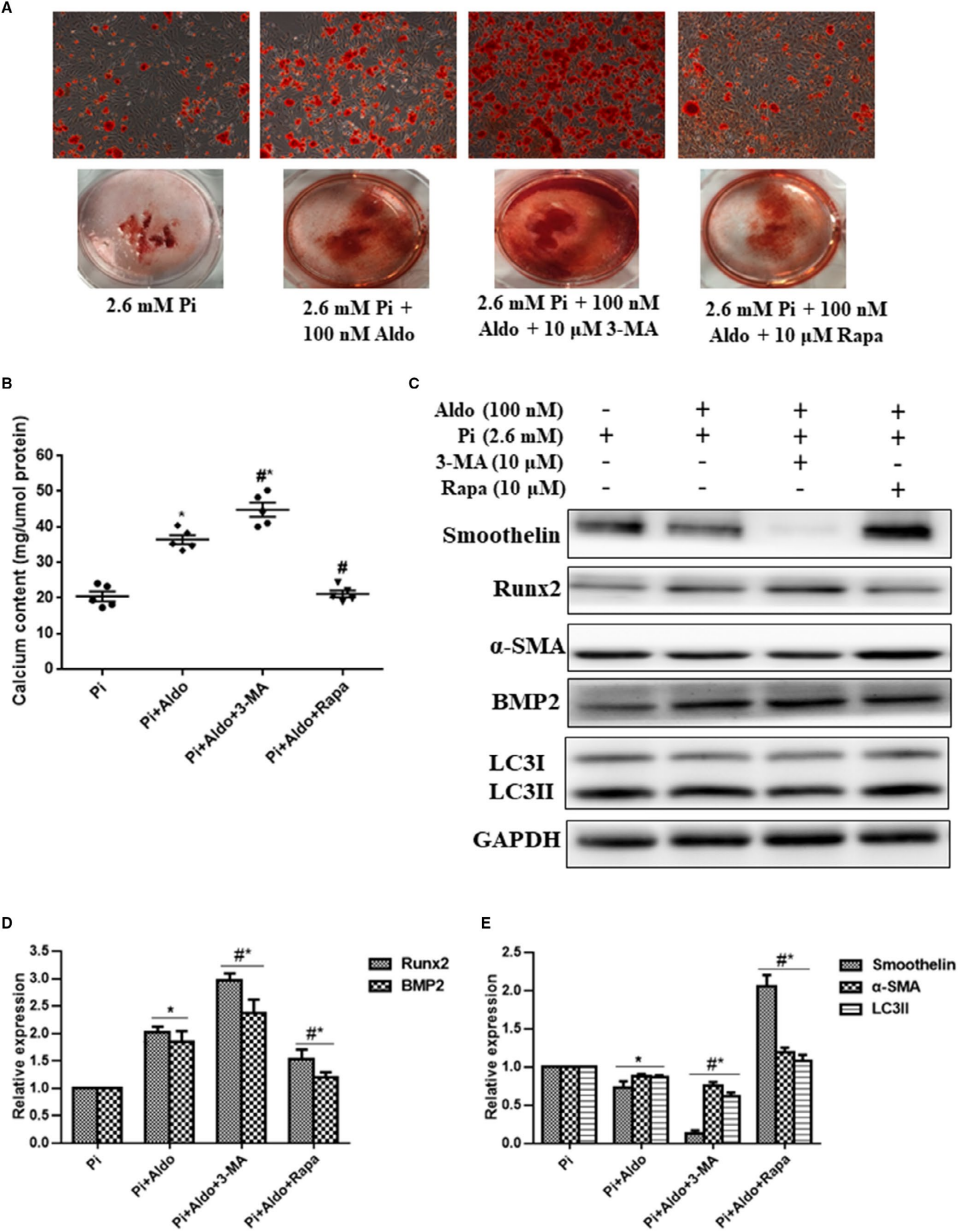
Aldosterone (Aldo) promoted calcification and osteogenic differentiation of vascular smooth muscle cells (VSMCs) via inhibition of autophagosomes. A, Alizarin red S staining. B, Quantitative analysis of the calcium content of VSMCs after induction with different stimuli for 7 d, normalized to the protein levels, and 100 nmol/L Aldo, 10 μmol/L 3‐methyladenine (3‐MA) and 10 μmol/L rapamycin (Rapa) were used. C‐E, Representative Western blot bands and semiquantitative analysis of LC3, smoothelin, α‐SMA, BMP2 and Runx2. n = 5 per group. *P < .05 vs control group; ^#^ *P* < .05 vs Pi group; ^&^ *P* < .05 vs Aldo + Pi group

Next, we aimed to investigate the role of autophagy in VSMC calcification and osteogenic differentiation aggravated by Aldo. As shown in Figure 4A,B, Aldo augmented high Pi‐induced VSMC calcification, and addition of the pharmacological inhibitor, 3‐MA (10 μmol/L), further accelerated this effect. However, treatment of the pharmacological inducer, Rapa (10 μmol/L), significantly attenuated Aldo‐enhanced VSMC calcification (*P* < .05). Pre‐treating VSMCs with 3‐MA further aggravated LC3II down‐regulation, whereas Rapa treatment abrogated LC3II reduction induced by Aldo. Simultaneously, 3‐MA augmented and Rapa inhibited the osteogenic differentiation of VSMCs enhanced by Aldo (Figure 4C,D; *P* < .05).

**FIGURE 5:**
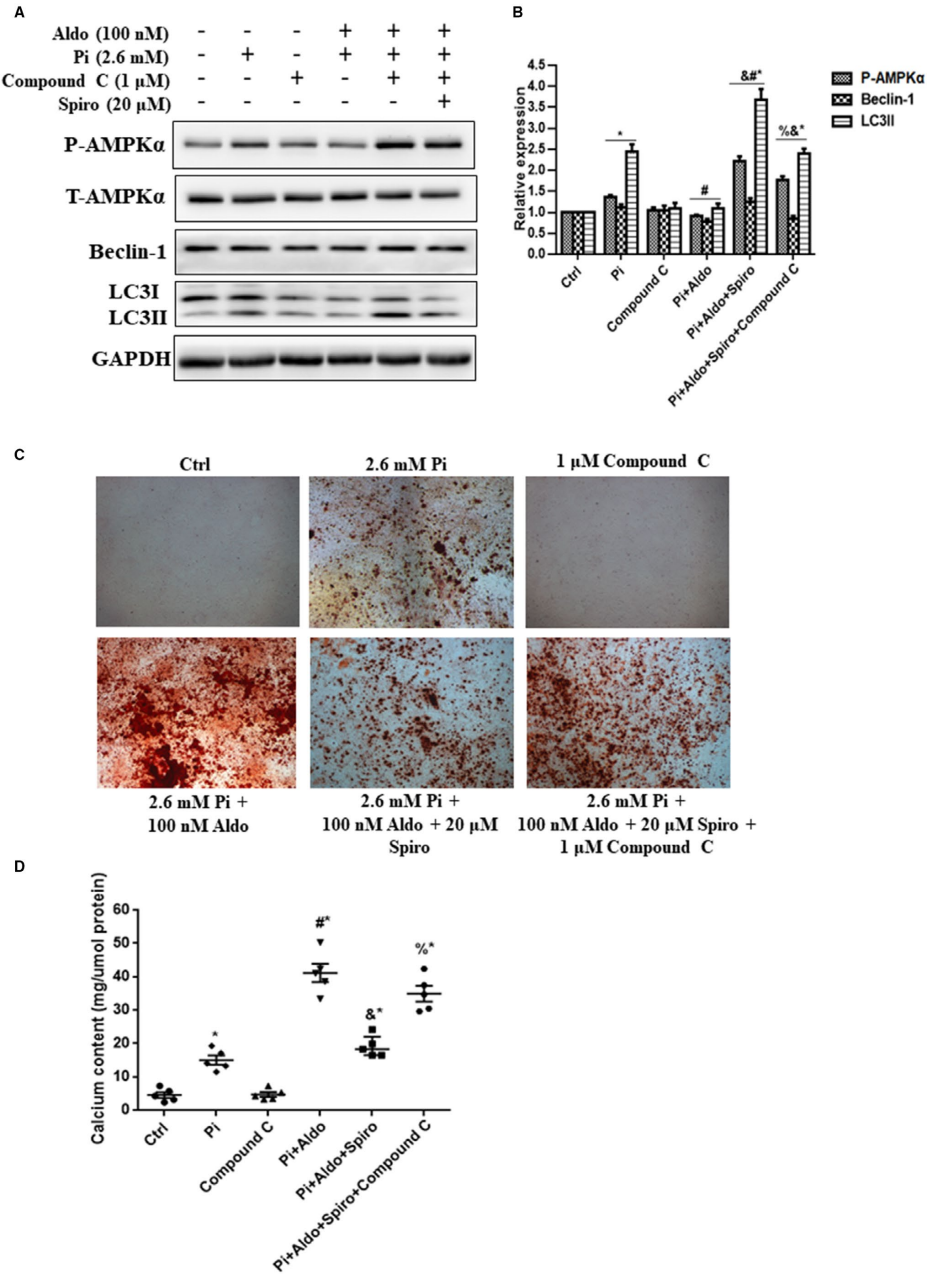
Adenosine monophosphate‐activated protein kinase (AMPK) phosphorylation mediated aldosterone (Aldo)‐mineralocorticoid receptor‐dependent inhibition of vascular smooth muscle cells (VSMC) autophagy. A and B, Representative Western blot bands and semiquantitative analysis of LC3, Beclin‐1, phospho‐AMPKα (P‐AMPKα) and total AMPKα (T‐AMPKα) in VSMCs. C, Alizarin red S staining of microscopic (scale bar, 50 μm) images in mice VSMCs. D, Quantitative analysis of the calcium content of VSMCs after induction with different stimuli for 7 d, normalized to the protein levels, and 100 nmol/L Aldo, 1 µmol/L Compound C and 20 μmol/L spironolactone (Sipro) were used. n = 5 per group. *P < .05 vs control group; ^#^ *P* < .05 vs Pi group; ^&^ *P* < .05 vs Aldo + Pi group; ^%^ *P* < .05 vs Aldo + Pi + Spiro group

As AMPK signalling was reported as an important regulator of autophagy,^15^ we next investigated the role of AMPK signalling in the Aldo‐MR complex‐mediated inhibition of VSMC autophagy. As shown in Figure 5A,B, addition of Aldo reduced the ratio of P‐AMPKα increased by high Pi, accompanied by down‐regulation of LC3II and Beclin‐1 protein expression. This effect was mimicked by AMPK inhibitor, compound C (1 μmol/L), but was reversed by cotreatment with Spiro (*P* < .05). Consistently, the protective effect of Spiro on Aldo‐enhanced VSMC calcification was blocked by compound C (Figure 5C,D; *P* < .05).

## 4 DISCUSSION

Our present study demonstrates that only in the calcified mellitus induced by high Pi medium, Aldo binds to mineralocorticoid receptor (MR) and induces a switch of VSMCs from contractile to osteogenic phenotype, promoting VSMC calcification. The molecular mechanisms underlying these processes involve AMPK‐dependent autophagosome formation.

Previous studies have indicated the association between Aldo excess and VC, but the role of high Pi in this association has been debated.^6^, ^7^, ^8^, ^9^, ^28^, ^29^ Some investigators found that only in calcified mellitus induced by high Pi, Aldo promoted VC through a MR‐dependent manner.^7^, ^8^ In addition, inhibition of type III sodium‐dependent Pi cotransporter Pit‐1 significantly abrogated the procalcific effects of Aldo, suggesting that it required high Pi mellitus for Aldo‐enhanced VC.^9^ However, some argued that long‐term exposure to Aldo alone could induce VSMC calcification.^6^, ^28^, ^29^ In this study, we used DBA/2N mice to build VC model by feeding 3 weeks' HPD. Although the phosphorus concentration in diet (2%) in standard regimen for building VC in these mice was much higher than that was consumed by humans,^20^, ^30^ this model did not affect the kidney function; in turn, potential confounding effects of other VC promoters that were commonly elevated in advanced CKD were excluded.^10^, ^20^, ^31^ Thus, it was suitable for us to investigate the independent role of Aldo in VC. Our in vivo experiments for the first time showed that chronic Aldo exposure (DOCA) alone did not induce VC in DBA/2N mice with standard chow containing normal Pi, but DOCA significantly accelerated the VC in DBA/2N mice with HPD. Results from in vitro study consistently showed that Aldo excess only enhanced VSMC calcification in the presence of high Pi. Thus, these findings indicate that Aldo is a strong VC promoter rather than an initiator.

VC is considered as an actively regulated process resembling bone formation. In calcified blood vessels, bone‐related transcription factors, including Msx2, Runx2 and osterix, have been detected in VSMCs. BMPs can activate Msx2 and Wnt signalling to up‐regulate Runx2 and osterix expression.^26^ Runx2 and osterix, in turn, increase expression of the bone‐related proteins including OCN, sclerostin and osteopontin. These changes finally result in a phenotypic conversion of VSMCs from a contractile to an osteoblastic phenotype. Once osteoblast‐like cells express alkaline phosphatase, they synthesize hydroxyapatite crystals.^26^ This reverse change of arterial and skeletal mineralization is proposed as bone‐vascular axis,^32^ indicating that bone loss may predict VC formation. Bone metabolism was reported to be affected by Aldo‐MR–mediated effects on osteoblasts and osteoclasts.^33^ Several clinical studies showed that patients with PA had reduced bone mineral density and increased risk of vertebral fracture.^34^, ^35^ We recently provided the first human data that hyperaldosteronism was closely associated with abdominal aortic calcification.^11^ In the present study, we further demonstrated that Aldo promoted high Pi‐induced phenotypic switch of VSMCs, characterized by up‐regulation of osteogenic phenotype markers (BMP2, Runx2 and OCN) and down‐regulation of contractile phenotype markers (α‐SMA, SM22α and smoothelin) in a MR‐dependent way.

Emerging evidence indicates that VSMC autophagy is a potential therapeutic target for VC.^20^ Moderate up‐regulation of VSMC autophagy was reported to inhibit VSMC osteogenic differentiation by degrading Runx2.^36^ Previous studies demonstrated an association between Aldo excess and autophagy in different tissues and cells except for vasculature.^14^, ^15^, ^16^ In the present study, we showed for the first time that VSMC autophagosomes formation was inhibited in the Aldo‐enhanced calcification. In addition, the pharmacological inhibitor of autophagy 3‐MA demonstrated a cumulative stimulatory effect of Aldo on the VSMC osteogenic differentiation and calcification. By contrast, the autophagy inducer, rapamycin attenuated VSMC calcification promoted by Aldo. Moreover, we revealed that AMPK signalling‐dependent VSMC autophagy was a potential molecular mechanism for Aldo/MR‐mediated calcification in VSMCs.

Multiple mechanisms are involved in VC,^37^ such as inflammation, oxidative stress and VSMC apoptosis. Whether these processes are also involved in Aldo‐enhanced VC still needs further investigation. Additionally, although we showed that MR‐dependent mechanism played a key role in Aldo procalcific effects, we could not exclude the MR‐independent effects in Aldo‐increased VC.^38^ Therefore, further studies are warranted.

**FIGURE 6:**
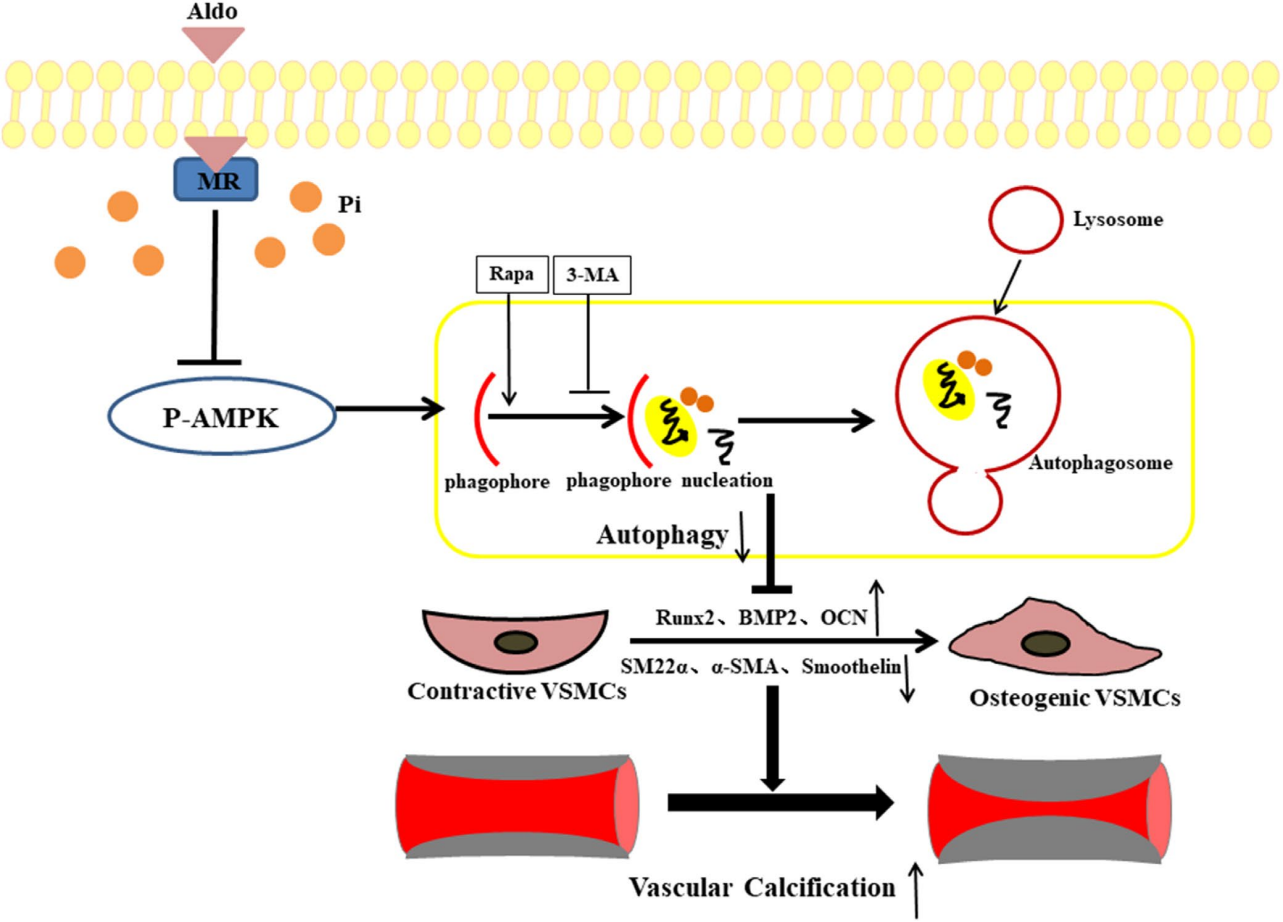
Schematic representation of the molecular mechanism for aldosterone (Aldo)‐enhanced vascular smooth muscle cell (VSMC) calcification. In the presence of high phosphate (Pi), Aldo binds to mineralocorticoid receptor (MR) in cytoplasm and inhibits adenosine monophosphate‐activated protein kinase (AMPK) phosphorylation, which then retards formation of VSMC autophagosome, in turn promotes phenotypic switch of VSMCs from the contractile to the osteogenic form, leading to enhanced VSMC calcification

In conclusion, we identified Aldo as a promoter of VC, rather than an initiator. Under calcified conditions, Aldo excess facilitated VSMC osteogenic differentiation and subsequent calcification via inhibition of AMPK‐dependent autophagy (Figure 6). Inhibition of Aldo/MR signalling by MR blockers or pharmacological up‐regulation of autophagy may protect against VSMC osteogenesis and VC in the setting of Aldo excess.

## CONFLICT OF INTEREST

The authors confirm that there are no conflicts of interest.

## AUTHOR CONTRIBUTIONS

**Jing‐Wei Gao:** Conceptualization (equal); data curation (equal); formal analysis (lead); funding acquisition (equal); investigation (equal); methodology (equal); project administration (lead); validation (equal); writing – original draft (lead); writing – review & editing (lead). **Wan‐Bing He:** Data curation (equal); investigation (equal); methodology (equal); validation (equal). **Chang‐Ming Xie:** Data curation (equal); investigation (equal); methodology (equal); validation (equal). **Ming Gao:** Software (equal). **Lei‐Yu Feng:** Methodology (equal); resources (equal). **Zhao‐Yu Liu:** Writing – review & editing (equal). **Jing‐Feng Wang:** Supervision (equal). **Hui Huang:** Funding acquisition (equal); supervision (equal). **Pin‐Ming Liu:** Funding acquisition (equal); supervision (equal).

## Supporting information

Fig S1‐3

## Data Availability

The data sets generated and/or analysed during the current study are available from the corresponding author on reasonable request.

## References

[1] Liabeuf S , Okazaki H , Desjardins L , et al. Vascular calcification in chronic kidney disease: are biomarkers useful for probing the pathobiology and the health risks of this process in the clinical scenario? Nephrol Dial Transplant. 2014;29:1275‐1284. 24009287 10.1093/ndt/gft368

[2] Lee SJ , Lee IK , Jeon JH . Vascular calcification‐new insights into its mechanism. Int J Mol Sci. 2020;21:2685. 10.3390/ijms21082685 PMC7216228 32294899

[3] Ferrario CM , Mullick AE . Renin angiotensin aldosterone inhibition in the treatment of cardiovascular disease. Pharmacol Res. 2017;125:57‐71. 28571891 10.1016/j.phrs.2017.05.020 PMC5648016

[4] McCurley A , Jaffe IZ . Mineralocorticoid receptors in vascular function and disease. Mol Cell Endocrinol. 2012;350:256‐265. 21723914 10.1016/j.mce.2011.06.014 PMC3214604

[5] Monticone S , D'Ascenzo F , Moretti C , et al. Cardiovascular events and target organ damage in primary aldosteronism compared with essential hypertension: a systematic review and meta‐analysis. Lancet Diabetes Endocrinol. 2018;6:41‐50. 29129575 10.1016/S2213-8587(17)30319-4

[6] Jaffe IZ , Tintut Y , Newfell BG , Demer LL , Mendelsohn ME . Mineralocorticoid receptor activation promotes vascular cell calcification. Arterioscler Thromb Vasc Biol. 2007;27:799‐805. 17234727 10.1161/01.ATV.0000258414.59393.89

[7] Tatsumoto N , Yamada S , Tokumoto M , et al. Spironolactone ameliorates arterial medial calcification in uremic rats: the role of mineralocorticoid receptor signaling in vascular calcification. Am J Physiol Renal Physiol. 2015;309:F967‐F979. 26336165 10.1152/ajprenal.00669.2014

[8] Wu SY , Yu YR , Cai Y , et al. Endogenous aldosterone is involved in vascular calcification in rat. Exp Biol Med (Maywood). 2012;237:31‐37. 22185918 10.1258/ebm.2011.011175

[9] Voelkl J , Alesutan I , Leibrock CB , et al. Spironolactone ameliorates Pit1‐dependent vascular osteoinduction in klotho‐hypomorphic mice. J Clin Invest. 2013;123:812‐822. 23298834 10.1172/JCI64093 PMC3561808

[10] Schlieper G , Schurgers L , Brandenburg V , Reutelingsperger C , Floege J . Vascular calcification in chronic kidney disease: an update. Nephrol Dial Transplant. 2016;31:31‐39. 25916871 10.1093/ndt/gfv111

[11] Lang F , Ritz E , Voelkl J , Alesutan I . Vascular calcification–is aldosterone a culprit? Nephrol Dial Transplant. 2013;28:1080‐1084. 23476041 10.1093/ndt/gft041

[12] Liu P , Zhang S , Gao J , et al. Downregulated serum 14, 15‐epoxyeicosatrienoic acid is associated with abdominal aortic calcification in patients with primary aldosteronism. Hypertension. 2018;71:592‐598. 29440332 10.1161/HYPERTENSIONAHA.117.10644

[13] Cazaña‐Pérez V , Cidad P , Donate‐Correa J , et al. Phenotypic modulation of cultured primary human aortic vascular smooth muscle cells by uremic serum. Front Physiol. 2018;9:89. 29483881 10.3389/fphys.2018.00089 PMC5816230

[14] Bai M , Che R , Zhang Y , et al. Reactive oxygen species‐initiated autophagy opposes aldosterone‐induced podocyte injury. Am J Physiol Renal Physiol. 2016;310:F669‐F678. 26764202 10.1152/ajprenal.00409.2015

[15] Luo X , Wang D , Luo X , et al. Caveolin 1‐related autophagy initiated by aldosterone‐induced oxidation promotes liver sinusoidal endothelial cells defenestration. Redox Biol. 2017;13:508‐521. 28734243 10.1016/j.redox.2017.07.011 PMC5521033

[16] Wang L , Mao N , Tan RZ , et al. Ginsenoside Rg1 reduces aldosterone‐induced autophagy via the AMPK/mTOR pathway in NRK‐52E cells. Int J Mol Med. 2015;36:518‐526. 26063203 10.3892/ijmm.2015.2242

[17] Vindis C . Autophagy: an emerging therapeutic target in vascular diseases. Br J Pharmacol. 2015;172:2167‐2178. 25537552 10.1111/bph.13052 PMC4403086

[18] Tai S , Hu XQ , Peng DQ , Zhou SH , Zheng XL . The roles of autophagy in vascular smooth muscle cells. Int J Cardiol. 2016;211:1‐6. 26954728 10.1016/j.ijcard.2016.02.128

[19] Dai XY , Zhao MM , Cai Y , et al. Phosphate‐induced autophagy counteracts vascular calcification by reducing matrix vesicle release. Kidney Int. 2013;83:1042‐1051. 23364520 10.1038/ki.2012.482

[20] Phadwal K , Feng D , Zhu D , MacRae VE . Autophagy as a novel therapeutic target in vascular calcification. Pharmacol Ther. 2020;206:107430. 31647975 10.1016/j.pharmthera.2019.107430

[21] Frauscher B , Kirsch AH , Schabhüttl C , et al. Autophagy protects from uremic vascular media calcification. Front Immunol. 2018;9:1866. 30154792 10.3389/fimmu.2018.01866 PMC6102358

[22] Hernandez ME , Watkins JM , Vu J , Hayward LF . DOCA/salt hypertension alters Period1 and orexin‐related gene expression in the medulla and hypothalamus of male rats: diurnal influences. Auton Neurosci. 2018;210:34‐43. 29246398 10.1016/j.autneu.2017.12.003

[23] Zhou P , Zhang X , Guo M , et al. Ginsenoside Rb1 ameliorates CKD‐associated vascular calcification by inhibiting the Wnt/β‐catenin pathway. J Cell Mol Med. 2019;23:7088‐7098. 31423730 10.1111/jcmm.14611 PMC6787443

[24] Yang R , Zhu Y , Wang Y , et al. HIF‐1α/PDK4/autophagy pathway protects against advanced glycation end‐products induced vascular smooth muscle cell calcification. Biochem Biophys Res Commun. 2019;517:470‐476. 31376939 10.1016/j.bbrc.2019.07.102

[25] Zeng JW , Zeng XL , Li FY , et al. Cystic Fibrosis Transmembrane Conductance Regulator (CFTR) prevents apoptosis induced by hydrogen peroxide in basilar artery smooth muscle cells. Apoptosis. 2014;19:1317‐1329. 24999019 10.1007/s10495-014-1014-z

[26] Leopold JA . Vascular calcification: mechanisms of vascular smooth muscle cell calcification. Trends Cardiovasc Med. 2015;25:267‐274. 25435520 10.1016/j.tcm.2014.10.021 PMC4414672

[27] Salabei JK , Cummins TD , Singh M , Jones SP , Bhatnagar A , Hill BG . PDGF‐mediated autophagy regulates vascular smooth muscle cell phenotype and resistance to oxidative stress. Biochem J. 2013;451:375‐388. 23421427 10.1042/BJ20121344 PMC4040966

[28] Alesutan I , Voelkl J , Feger M , et al. Involvement of vascular aldosterone synthase in phosphate‐induced osteogenic transformation of vascular smooth muscle cells. Sci Rep. 2017;7:2059. 28515448 10.1038/s41598-017-01882-2 PMC5435689

[29] Hao J , Zhang L , Cong G , Ren L , Hao L . MicroRNA‐34b/c inhibits aldosterone‐induced vascular smooth muscle cell calcification. Cell Tissue Res. 2016;366:733‐746. 27503378 10.1007/s00441-016-2469-8

[30] Yoshida T , Yamashita M , Horimai C , Hayashi M . Smooth muscle‐selective nuclear factor‐κB inhibition reduces phosphate‐induced arterial medial calcification in mice with chronic kidney disease. J Am Heart Assoc. 2017;6:e007248. 29146611 10.1161/JAHA.117.007248 PMC5721793

[31] Shiozaki Y , Okamura K , Kohno S , et al. The CDK9‐cyclin T1 complex mediates saturated fatty acid‐induced vascular calcification by inducing expression of the transcription factor CHOP. J Biol Chem. 2018;293:17008‐17020. 30209133 10.1074/jbc.RA118.004706 PMC6222109

[32] Thompson B , Towler DA . Arterial calcification and bone physiology: role of the bone‐vascular axis. Nat Rev Endocrinol. 2012;8:529‐543. 22473330 10.1038/nrendo.2012.36 PMC3423589

[33] Tomaschitz A , Ritz E , Pieske B , et al. Aldosterone and parathyroid hormone interactions as mediators of metabolic and cardiovascular disease. Metabolism. 2014;63:20‐31. 24095631 10.1016/j.metabol.2013.08.016

[34] Petramala L , Zinnamosca L , Settevendemmie A , et al. Bone and mineral metabolism in patients with primary aldosteronism. Int J Endocrinol. 2014;2014:836529. 24864141 10.1155/2014/836529 PMC4016829

[35] Notsu M , Yamauchi M , Yamamoto M , Nawata K , Sugimoto T . Primary aldosteronism as a risk factor for vertebral fracture. J Clin Endocrinol Metab. 2017;102:1237‐1243. 28182819 10.1210/jc.2016-3206

[36] Peng YQ , Xiong D , Lin X , et al. Oestrogen inhibits arterial calcification by promoting autophagy. Sci Rep. 2017;7:3549. 28615727 10.1038/s41598-017-03801-x PMC5471178

[37] Johnson RC , Leopold JA , Loscalzo J . Vascular calcification: pathobiological mechanisms and clinical implications. Circ Res. 2006;99:1044‐1059. 17095733 10.1161/01.RES.0000249379.55535.21

[38] Gao J , Zhang K , Chen J , et al. Roles of aldosterone in vascular calcification: an update. Eur J Pharmacol. 2016;786:186‐193. 27238972 10.1016/j.ejphar.2016.05.030

