# Supplementary material for: Aldosterone enhances high phosphate–induced vascular calcification through inhibition of AMPK‐mediated autophagy
Source: J Cell Mol Med. 2020 Nov 4;24(23):13648–59. doi: 10.1111/jcmm.15813 (PMC7754028; doi:10.1111/jcmm.15813)

**Supplemental Figure S1** Primary mouse VSMC identification immunostained for α-SMA (green).

**
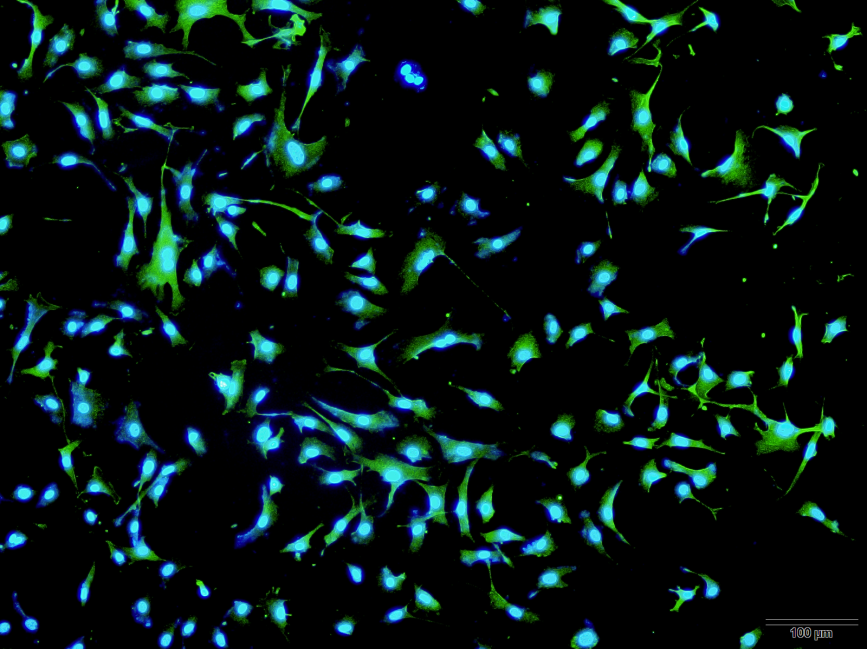
**

**Supplemental Figure S2** Dose-dependent experiments of NaH2PO4 (Pi) on VSMC calcification measured by Alizarin red S staining.


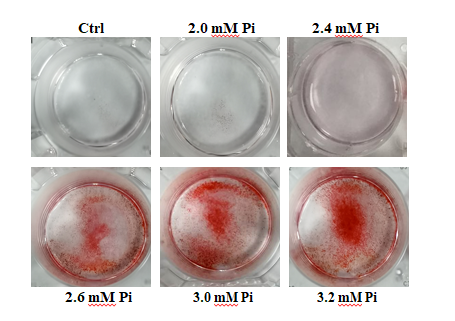


**Supplementary Figure S3** Effects of different multiplicity of the mRFP-GFP adenovirus on VSMC viability measured by CCK-8 (^*^*P*<0.05 vs. control).


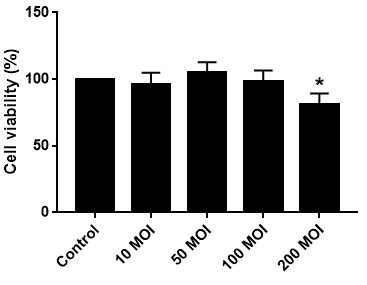

Supplement: Supplementary file 1 — Fig S1‐3 [file JCMM-24-13648-s001.docx]
